# Comparison of complications with a 1.25-mm versus a 1.5-mm burr for severely calcified lesions that could not be crossed by an intravascular ultrasound catheter

**DOI:** 10.1007/s12928-019-00606-9

**Published:** 2019-07-20

**Authors:** Kenichi Sakakura, Yousuke Taniguchi, Kei Yamamoto, Takunori Tsukui, Masaru Seguchi, Hiroshi Wada, Shin-ichi Momomura, Hideo Fujita

**Affiliations:** Division of Cardiovascular Medicine, Saitama Medical Center, Jichi Medical University, 1-847 Amanuma, Omiya, Saitama, 330-8503 Japan

**Keywords:** Rotational atherectomy, Intravascular ultrasound, Slow flow, Percutaneous coronary intervention, Complications

## Abstract

Since intravascular imaging such as intravascular ultrasound (IVUS) can provide useful information for rotational atherectomy (RA), intravascular imaging should be attempted before RA. However, some calcified lesions do not allow imaging catheters to cross before RA. Although small burrs (1.25 mm or 1.5 mm) should be selected for such tight lesions, it is unknown whether a 1.25-mm burr or 1.5-mm burr is safer as the initial burr. The aim of this study was to compare the incidence of complications with a 1.25-mm versus a 1.5-mm burr as the initial burr for IVUS-uncrossable lesions. This was a retrospective, single-center study. A total of 109 IVUS-uncrossable lesions were included, and were divided into a 1.25-mm group (*n *=52) and a 1.5-mm group (*n *=57). The incidence of slow flow just after RA was not different between the 2 groups (1.25-mm group: 25%, 1.5-mm group: 31.6%, *P *=0.45). The incidence of peri-procedural MI with slow flow was not different and equally low in the 2 groups (1.25-mm group: 1.9%, 1.5-mm group: 3.5%, *P *=0.61). The use of the 1.5-mm burr as the initial burr was not significantly associated with slow flow after controlling for chronic renal failure on hemodialysis and reference diameter (vs. 1.25-mm: OR 2.34, 95% CI 0.89–6.19, *P *=0.09). In conclusion, the incidence of complications following RA was comparable between the 1.25-mm and the 1.5-mm burrs as the initial burr for IVUS-uncrossable lesions. The present study provides insights into the selection of an appropriate burr for IVUS-uncrossable lesions.

## Introduction

Although rotational atherectomy (RA) is a crucial device for severely calcified coronary lesions, severe complications such as type III perforation is more frequently observed in percutaneous coronary interventions (PCI) with than without RA [1, 2]. Because intravascular imaging devices such as intravascular ultrasound (IVUS) or optical coherence tomography (OCT) can provide additional information regarding calcification beyond angiography [3–6], the use of intravascular imaging devices may help operators to avoid serious complications during RA. In fact, operators can choose an appropriate initial burr (1.25 mm, 1.5 mm, or ≥ 1.75 mm) or RotaWire (floppy or extra-support type) based on IVUS or OCT images [7]. Moreover, since current imaging devices are low profile [8], it is reasonable to attempt intravascular imaging before RA.

However, some severely calcified lesions do not allow intravascular imaging devices to cross before RA [9]. If low-profile imaging devices cannot cross the lesion, the initial burr size should be either the smallest burr (1.25 mm) or the second smallest burr (1.5 mm) to avoid serious complications. In general, a smaller device seems to be more useful than a bigger device for severely calcified lesion [10]. However, the shape of each RA burr is close to an ellipsoid [11]. While the short axes are different between the burrs, the long axes are approximately the same [11]. Therefore, the smallest burr (1.25 mm) is the sharpest ellipsoid, which may be associated with complications such as burr entrapment or perforation [12], and it is unknown whether a 1.25-mm burr or 1.5-mm burr is safer as an initial burr for severely calcified lesions that an intravascular imaging catheter cannot cross. The aim of the present study was to compare the incidence of complications with 1.25-mm versus 1.5-mm burrs for severely calcified lesions that an IVUS catheter could not cross.

## Methods

### Study design

**Fig. 1:**
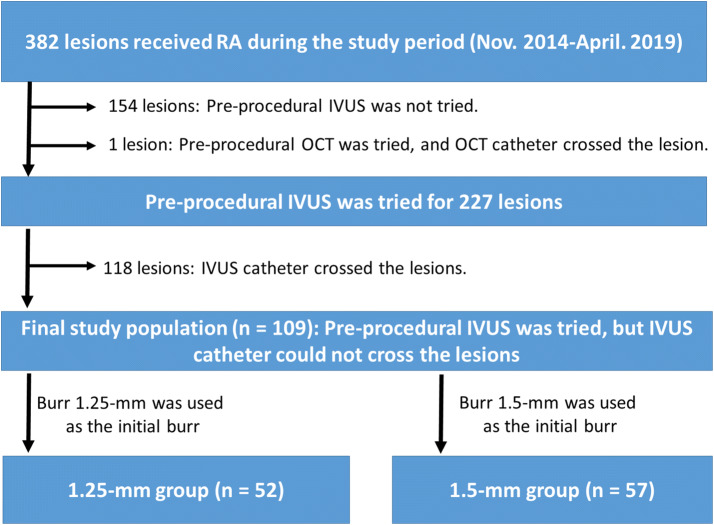
Study flow chart. *RA* rotational atherectomy, *IVUS* intravascular ultrasound, *OCT* optical coherence tomography

This was a retrospective, single-center study. We reviewed 382 consecutive coronary lesions that were treated by RA in our institution during the period from November 2014 to April 2019. Indications for RA in our institution are the following: [1] angiographically moderate or severely calcified lesions, [2] diffuse lesions expected to be difficult to stent, and [3] ostial lesions [13, 14]. We excluded 154 lesions in which pre-procedural IVUS was not attempted, and also excluded one lesion in which pre-procedural OCT was attempted but the OCT catheter could cross the lesion. Among 227 lesions in which pre-procedural IVUS was attempted before RA, 118 lesions were excluded, because an IVUS catheter crossed the lesion before RA. The final study consisted of 109 lesions, in which an IVUS catheter could not cross the lesion before RA. The lesions were further classified according to the initial burr size (1.25 mm or 1.5 mm). The 109 lesions were divided into a 1.25-mm group (*n *=52) and a 1.5-mm group (*n *=57). The study flow chart is shown in Fig. 1. The study was approved by the institutional review board, and written informed consent was waved because of the retrospective study design.

### Rotational atherectomy

RA was performed using standard techniques, which was described in our previous publications [14]. A nicorandil-based drug cocktail (nicorandil 12 mg, isosorbide dinitrate 2.5 mg, heparin 10,000 units, and normal saline 500 mL) was used during RA to prevent slow flow [15, 16]. The lesion was crossed with a 0.014-inch conventional guidewire, and IVUS was attempted. The type of IVUS catheter was selected based on the discretion of the interventional cardiologist. Among 109 lesions, OptiCross™ (*n *=76) (Boston Scientific, Marlborough, MA, USA), Navifocus^Ⓡ^ WR (*n *=24) (Terumo, Tokyo, Japan), AltaView^Ⓡ^ (*n *=9) (Terumo, Tokyo, Japan), and Eagle Eye^Ⓡ^ (*n *=3) (Phillips Volcano, San Diego, CA, USA) were used as the IVUS catheter. After the failure of IVUS, a 0.014-inch conventional guidewire was exchanged with a 0.009-inch RotaWire floppy or RotaWire extra support guidewire (Boston Scientific, Marlborough, MA, USA) using a microcatheter. The RA burr was subsequently advanced over the wire to a position proximal to the lesion. The initial rotational speed was set within the conventional range (140,000–190,000 rpm) with the burr proximal to the lesion, and several lesions were randomly allocated to 140,000 rpm or 190,000 rpm [14]. The burr was activated and moved forward with a slow pecking motion. Each run time was < 30 s, and care was taken to avoid a decrease in rotational speed > 5000 rpm. The initial burr size was either 1.25 mm or 1.5 mm, which is supported by the European expert consensus on RA [17]. In most cases, the selection of the initial burr size was at the discretion of a senior interventional cardiologist (K. Sakakura). After the burr passed the lesion, the burr was removed using the dynaglide mode or trapping balloon technique [18]. The presence of coronary flow was confirmed by injecting sufficient contrast medium immediately after the burr was removed. Following RA, balloon dilatation was performed using a non-compliant balloon to facilitate stent implantation.

RA was not used as first-line therapy to treat culprit lesions in acute coronary syndrome (ACS); however, RA was used to treat culprit lesions in ACS if necessary [15]. Furthermore, an intra-aortic balloon pump (IABP) was inserted via a femoral artery before RA in high-risk cases such as those with severe left ventricular dysfunction, unprotected left main stenosis, or severe 3-vessel disease.

### Complications

We collected data on the following complications: slow flow just after RA, thrombolysis in myocardial infarction (TIMI) flow grade just after RA, vessel perforation (type III) due to the burr, and peri-procedural myocardial infarction with slow flow. Slow flow just after RA was defined as slow or absent distal runoff (TIMI flow grade ≤ 2) [15, 16]. Peri-procedural myocardial infarction was defined as an increase in creatine kinase (at least threefold above the normal upper limit) [13, 14].

### Definitions

eGFR was calculated using the MDRD formula [19]. ACS was defined as ST-segment elevation myocardial infarction, non-ST-segment elevation myocardial infarction, or unstable angina. The reference diameter and lesion length were calculated by quantitative coronary angiography. Offline, computer-based software QAngio XA 7.3 (MEDIS Imaging Systems, Leiden, The Netherlands) was used for quantitative coronary angiography. The burr–artery ratio was defined as the burr size divided by the reference diameter.

### Statistical analysis

Data are presented as a percentage for categorical variables and the mean ± SD for continuous variables. The Wilk–Shapiro test was performed to determine if the continuous variables were normally distributed. Normally distributed continuous variables were compared between the 2 groups using a Student’s *t* test. Otherwise, continuous variables were compared using a Mann–Whitney U test. Categorical data were compared using a Chi-square test. We performed multivariate logistic regression analysis to investigate the association between the initial burr size and slow flow. In this model, the dependent variable was slow flow just after RA. Variables that had a significant association (*P *< 0.05) between the 2 groups were used as independent variables. All variables were simultaneously adjusted in one step. Odds ratios (OR) and the 95% confidence intervals (CI) were calculated. All reported *P* values were determined by two-sided analysis, and *P* values < 0.05 were considered significant. All analyses were performed with IBM SPSS statistics version 25 (Chicago, IL, USA).

## Results

The comparison of patients and lesion characteristics between the 2 groups is summarized in Table 1. The patient characteristics were comparable except for the prevalence of chronic renal failure on hemodialysis, which was significantly greater in the 1.5-mm group (31.6%) than the 1.25-mm group (15.4%) (*P *=0.048). The lesion characteristics were also comparable except for the reference diameter, which was significantly greater in the 1.5-mm group (2.36 ± 0.57 mm) than the 1.25-mm group (2.15 ± 0.62 mm) (*P *=0.03).

**Table 1:** Comparison of patients and lesions characteristics between the 1.25-mm group and 1.5-mm group

	All (*n *=109)	1.25-mm group (*n *=52)	1.5-mm group (*n *=57)	*P* value
Patient characteristics				
Age (years)	73.2 ± 9.1	74.7 ± 8.3	71.9 ± 9.7	0.16
Men—*n*, (%)	71 (65.1)	33 (63.5)	38 (66.7)	0.73
Overweight (BMI ≥ 25 kg/m^2^)—*n*, (%)	34 (31.2)	16 (30.8)	18 (31.6)	0.93
Hypertension—*n*, (%)	107 (98.2)	51 (98.1)	56 (98.2)	0.95
Diabetes mellitus—*n*, (%)	68 (62.4)	29 (55.8)	39 (68.4)	0.17
Hyperlipidemia—*n*, (%)	102 (93.6)	48 (92.3)	54 (94.7)	0.61
Current smoker—*n*, (%)	13 (12.1)	6 (11.5)	7 (12.7)	0.85
Chronic renal failure (creatinine > 2 mg/dl)—*n*, (%)	31 (28.4)	13 (25.0)	18 (31.6)	0.45
Estimated GFR (mL/mn/1.73 m^2^)	62.5 ± 42.2	65.5 ± 40.9	59.9 ± 43.6	0.67
Chronic renal failure on hemodialysis—*n*, (%)	26 (23.9)	8 (15.4)	18 (31.6)	0.048
Statin treatment—*n*, (%)	100 (91.7)	46 (88.5)	54 (94.7)	0.23
Lesion characteristics				
Culprit lesion in acute coronary syndrome—*n*, (%)	29 (26.6)	14 (26.9)	15 (26.3)	0.94
Target coronary artery				0.62
Left main-left anterior descending artery—*n*, (%)	64 (58.7)	33 (63.5)	31 (54.4)	
Left circumflex artery—*n*, (%)	10 (9.2)	4 (7.7)	6 (10.5)	
Right coronary artery—*n*, (%)	35 (32.1)	15 (28.8)	20 (35.1)	
Specific target coronary artery				
Ostial left anterior descending artery—*n*, (%)	9 (8.3)	5 (9.6)	4 (7.0)	0.62
Ostial left circumflex artery—*n*, (%)	1 (0.9)	1 (1.9)	0 (0)	0.29
Ostial right coronary artery—*n*, (%)	9 (8.3)	4 (7.7)	5 (8.8)	0.84
Reference diameter (mm)	2.26 ± 0.60	2.15 ± 0.62	2.36 ± 0.57	0.03
Lesion length (mm)	27.37 ± 15.48	27.96 ± 14.71	26.83 ± 16.25	0.57

Data are expressed as the mean ± SD or number (percentage). A Mann–Whitney *U* test was used for continuous variables, and a Chi-square test was used for categorical variables

*GFR* glomerular filtration rate

The comparison of procedural characteristics between the 2 groups is summarized in Table 2. Because the number of burrs used were similar and approximately 1.3 burrs, the final burr size was significantly greater in the 1.5-mm group (1.52 ± 0.16 mm) than the 1.25-mm group (1.33 ± 0.20 mm) (*P *< 0.001). However, the final burr size was 1.25-mm in 5 lesions (8.8%) in the 1.5-mm group, indicating the burr was downsized in the 1.5-mm group. Moreover, other 5 lesions in the 1.5-mm group required the 1.25-mm burr during RA. Thus, a total of 10 lesions (17.5%) in the 1.5-mm group required downsize to the 1.25-mm burr. Other procedural characteristics were comparable between the 2 groups. Furthermore, when we divided the study lesions into the former 53 lesions (from November 2014 to December 2017) and the latter 56 lesions (from January 2018 to April 2019), the initial burr 1.5 mm was used in 16 lesions (30.2%) in the former period, and used in 41 lesions (73.2%) in the latter period.

**Table 2:** Comparison of procedural characteristics between the 1.25-mm group and 1.5-mm group

	All (*n *=109)	1.25-mm group (*n *=52)	1.5-mm group (*n *=57)	*P* value
Procedural characteristics				
Guiding catheter size and system				0.76
6Fr—*n*, (%)	3 (2.8)	2 (3.8)	1 (1.8)	
7Fr—*n*, (%)	101 (92.7)	48 (92.3)	53 (93.0)	
8Fr—*n*, (%)	5 (4.6)	2 (3.8)	3 (5.3)	
Intra-aortic balloon pump support—*n*, (%)	9 (8.3)	7 (13.5)	2 (3.5)	0.06
Guidewire used during rotational atherectomy				0.13
RotaWire floppy—*n*, (%)	78 (71.6)	39 (75.0)	39 (68.4)	
RotaWire extra support—*n*, (%)	12 (11.0)	2 (3.8)	10 (17.5)	
Guidewire switch from floppy to extra support—*n*, (%)	16 (14.7)	9 (17.3)	7 (12.3)	
Guidewire switch from extra support to floppy—*n*, (%)	3 (2.8)	2 (3.8)	1 (1.8)	
Number of burrs used	1.3 ± 0.5	1.3 ± 0.6	1.3 ± 0.4	0.35
Final burr size (mm)	1.43 ± 0.20	1.33 ± 0.20	1.52 ± 0.16	< 0.001
Final burr size as a categorical variable				< 0.001
1.25 mm	48 (44.0)	43 (82.7)	5 (8.8)	
1.5 mm	51 (46.8)	4 (7.7)	47 (82.5)	
1.75 mm	3 (2.8)	2 (3.8)	1 (1.8)	
2.0 mm	7 (6.4)	3 (5.8)	4 (7.0)	
Initial burr–artery ratio	0.65 ± 0.17	0.62 ± 0.16	0.67 ± 0.17	0.14
Final burr–artery ratio	0.67 ± 0.19	0.66 ± 0.20	0.68 ± 0.18	0.67
Total run time (seconds)	123.6 ± 85.8	119.9 ± 87.1	126.9 ± 85.2	0.61
Mean single run time (seconds)	14.2 ± 3.4	14.2 ± 4.1	14.1 ± 2.7	0.86
Mean rotational speed (× 1000 rpm)	174.6 ± 9.9	173.8 ± 11.4	175.3 ± 8.4	0.51
Maximum speed reduction during rotational atherectomy (rpm)	6701 ± 4003 (*n *=107)	6420 ± 4161 (*n *=50)	6947 ± 3880	0.16
Systolic blood pressure just before rotational atherectomy (mm Hg)	151.5 ± 24.9	152.2 ± 26.6	151.0 ± 23.4	0.80
Diastolic blood pressure just before rotational atherectomy (mm Hg)	75.0 ± 13.2	74.8 ± 14.7	75.1 ± 11.7	0.98
Heart rate just before rotational atherectomy (per minute)	72.9 ± 15.6	72.3 ± 14.2	73.5 ± 17.0	0.80
Final procedure				0.29
Rotational atherectomy + balloon—*n*, (%)	1 (0.9)	1 (1.9)	0 (0)	
Rotational atherectomy + bare-metal stent—*n*, (%)	1 (0.9)	0 (0)	1 (1.8)	
Rotational atherectomy + drug-eluting stent—*n*, (%)	100 (91.7)	49 (94.2)	51 (89.5)	
Rotational atherectomy + drug-eluting stent and drug-coated balloon—*n*, (%)	3 (2.8)	1 (1.9)	2 (3.5)	
Rotational atherectomy + covered stent for perforation—*n*, (%)	1 (0.9)	0 (0)	1 (1.8)	

Data are expressed as the mean ± SD or number (percentage). A Student’s *t* test or Mann–Whitney U test was used for continuous variables, and a Chi-square test was used for categorical variables

*GFR* glomerular filtration rate

The comparison of complications between the 2 groups is shown in Table 3. The incidence of slow flow just after RA was not different between the 2 groups. Furthermore, mean rotational speed was comparable between the patients with slow flow (177.0 ± 5.7 × 1000 rpm) and without slow flow (173.6 ± 11.1 × 1000 rpm) (*P *=0.15). The incidence of peri-procedural MI with slow flow was not different and equally low between the 2 groups. Because the use of IABP might affect the incidence of slow flow, we compared the incidence of slow flow between the 2 groups after excluding the patients with IABP. The incidence of slow flow remained comparable between the 1.25-mm group (26.7%) and the 1.5-mm group (30.9%) (*P *=0.64). A multivariate logistic regression model to investigate the association between the initial burr size and slow flow is shown in Table 4. A 1.5-mm burr as the initial burr was not significantly associated with slow flow after controlling for chronic renal failure on hemodialysis and reference diameter (vs. 1.25-mm: OR 2.34, 95% CI 0.89–6.19, *P *=0.09).

**Table 3:** Comparison of complications between the 1.25-mm and 1.5-mm groups

	All (*n *=109)	1.25-mm group (*n *=52)	1.5-mm group (*n *=57)	*P* value
Slow flow just after RA	31 (28.4)	13 (25.0)	18 (31.6)	0.45
TIMI flow grade just after RA				0.69
TIMI 1 flow	13 (11.9)	6 (11.5)	7 (12.3)	
TIMI 2 flow	18 (16.5)	7 (13.5)	11 (19.3)	
TIMI 3 flow	78 (71.6)	39 (75.0)	39 (68.4)	
Peri-procedural MI with slow flow	3 (2.8)	1 (1.9)	2 (3.5)	0.61
Vessel perforation (type III) due to the burr	1 (0.9)	1 (1.9)	0 (0)	0.29

Data are expressed as the number (percentage). A Chi-square test was used to compare the 2 groups

*TIMI* thrombolysis in myocardial infarction

**Table 4:** Multivariate logistic regression model to investigate the association between the initial burr size and slow flow

Independent variables	Odds ratio	95% confidence interval	*P* value
Dependent variable: slow flow			
1.5-mm burr as the initial burr (vs. 1.25-mm burr)	2.34	0.89–6.19	0.09
Chronic renal failure on hemodialysis (vs. non-hemodialysis)	0.86	0.27–2.70	0.79
Reference diameter (per 1-mm increase)	0.14	0.05–0.41	< 0.001

All variables were simultaneously adjusted in one step

## Discussion

A total of 109 severely calcified lesions that could not be crossed with pre-procedural IVUS catheters before RA were included in the present study, and were divided into a 1.25-mm group (*n *=52) and 1.5-mm group (*n *=57), according to the initial burr size. The incidence of complications such as slow flow just after RA or peri-procedural MI with slow flow were comparable between the 1.25-mm and 1.5-mm groups. A multivariate logistic regression analysis also confirmed that the use of a 1.5-mm burr as the initial burr was not associated with slow flow as compared with the use of a 1.25-mm burr as the initial burr. Our results suggest that the 1.25-mm and 1.5-mm burrs have similar performance with regard to the prevention of complications.

Since randomized studies in the 2000s showed better safety and similar efficacy with a small burr strategy (burr-to-artery ratio ≤ 0.70) compared with a large burr strategy (burr-to-artery ratio > 0.70) [20, 21], current expert opinions and consensus reports recommend the use of 1.25-mm or 1.5-mm burrs as the initial burr [17, 22], but the utility of intravascular imaging devices for RA was not fully discussed. Therefore, it would be practical to use a 1.25-mm or 1.5-mm burr for IVUS-uncrossable lesions. However, to the best of our knowledge, there are no studies that compare the performance or complications between the 1.25-mm and 1.5-mm burrs as the initial burr.

We should discuss why the incidence of complications was similar between the 1.25-mm and 1.5-mm burrs. One possible explanation is that both burr sizes were sufficiently small as an initial burr size for IVUS-uncrossable calcified lesions. If we had selected a 1.75-mm or 2.0-mm burr as an initial burr size for IVUS-uncrossable calcified lesions, we might have had more complications. Another explanation is that our operators might have selected a 1.25-mm burr for relatively smaller vessels, and selected a 1.5-mm burr for relatively larger vessels. Because the reference diameter was significantly greater in the 1.5-mm group than the 1.25-mm group, our operators tended to avoid the 1.5-mm burr for relatively smaller vessels. If we had randomly assigned IVUS-uncrossable lesions to a 1.25-mm or 1.5-mm burr, more complications might have been observed in the 1.5-mm group than in the 1.25-mm group.

We should mention the utility of the IVUS-uncrossable lesions as the indicator of calcified lesions that require RA. Although the contemporary objective of RA is plaque modification, the definite indication for RA is still lesions that any balloon cannot dilate or any devices including a microcatheter or the smallest balloons cannot cross. In fact, some IVUS-uncrossable calcified lesions may be crossed by small balloons and treated by balloon dilatation without RA. However, if balloon dilatation does not work, RA would be more difficult after balloon dilatation than before balloon dilatation, because balloon dilatation may provoke vessel dissection. Thus, it would be better to decide whether RA is necessary or not before balloon dilatation. On the other hand, since current low-profile IVUS catheters do not provoke vessel dissection, trying IVUS would not affect the procedural difficulty of RA. Therefore, it would be reasonable to perform RA to IVUS-uncrossable calcified lesions.

The clinical implications of the present study should be noted. Because the incidence of complications was not different between the 1.25-mm and 1.5-mm burrs, the bigger burr (1.5 mm) may be better as the initial burr for IVUS-uncrossable calcified lesions. Although the difference is only 0.25 mm between the 1.25-mm and 1.5-mm burrs, more calcification would be modified by the 1.5-mm burr than the 1.25-mm burr. Moreover, if operators have difficulty crossing the lesion with a 1.5-mm burr, they can downsize to a 1.25 mm, which is also recommended in the consensus report [17]. On the other hand, if operators have difficulty crossing the lesion with 1.25-mm burr, the next option is limited. Although upsizing to 1.5-mm burr may work for some lesions [9, 11], upsizing is not a standard strategy for the lesion that cannot be crossed with the initial burr.

### Study limitations

Because our study was designed as a single-center, retrospective, observational study, there is a risk of patient selection bias and group-selection bias. Especially, 1.5-mm burr was more frequently selected in the latter period (from January 2018 to April 2019) as compared to the former period (from November 2014 to December 2017). Because our team gained practical experience from each IVUS-uncrossable lesion, we tended to select the bigger burr (1.5 mm) in the latter period. Although the incidence of slow flow was relatively higher in our study than in earlier studies [16, 23], the incidence of peri-procedural MI with slow flow was comparable [23]. As we evaluated slow flow just after RA, most slow flow recovered immediately with or without intracoronary vasodilators, which should not cause peri-procedural MI. Although vessel perforation and burr entrapment are more serious and important complications than slow flow, our study population was too small to evaluate the difference in those important complications between the 2 groups. Finally, although the incidence of complications was not different between the 2 groups, there is a possibility of a beta error due to the small sample size [24].

## Conclusion

The incidence of complications following RA was comparable between the 1.25-mm and 1.5-mm burrs as the initial burr for IVUS-uncrossable severely calcified lesions. The 1.5-mm burr may be preferable as the initial burr, as long as the operators keep an option to downsize the 1.25-mm burr when they feel difficulty during RA. The present study provides insight into the selection of an appropriate burr for IVUS-uncrossable lesions.

## References

[1] Sakakura K, Inohara T, Kohsaka S, Amano T, Uemura S, Ishii H (2016). Incidence and determinants of complications in rotational atherectomy. Circ Cardiovasc Interv.

[2] Yamamoto S, Sakakura K, Funayama H, Wada H, Fujita H, Momomura S (2015). Percutaneous coronary artery bypass for type 3 coronary perforation. JACC Cardiovasc Interv.

[3] Maejima N, Hibi K, Saka K, Akiyama E, Konishi M, Endo M (2016). Relationship between thickness of calcium on optical coherence tomography and crack formation after balloon dilatation in calcified plaque requiring rotational atherectomy. Circ J.

[4] Yamamoto K, Sakakura K, Taniguchi Y, Wada H, Momomura SI, Fujita H (2017). A case of severely calcified neoatherosclerosis after paclitaxel eluting stent implantation. Cardiovas Revasc Med..

[5] Shiraishi J, Ohshiro M, Matsubara Y, Hyogo M, Uchiyama H, Sawada T (2018). Optical frequency domain imaging-guided rotational atherectomy followed by drug-coated balloon dilation to the non-calcified lesion in a patient with severe thrombocytopenia. Cardiovasc Interv Ther..

[6] Furuse E, Tanabe J, Tajiri M, Kawanaka H, Shimizu W (2019). In-stent restenosis caused by calcified nodule 11 years after paclitaxel eluting stent implantation treated with drug-coated balloon following rotational atherectomy. Cardiovasc Interv Ther..

[7] Sakakura K, Yamamoto K, Taniguchi Y, Tsurumaki Y, Momomura S-I, Fujita H (2018). Intravascular ultrasound enhances the safety of rotational atherectomy. Cardiovasc Revasc Med..

[8] Okamura A, Iwakura K, Fujii K (2010). ViewIT improves intravascular ultrasound-guided wiring in coronary intervention of chronic total occlusion. Catheter Cardiovasc Interv..

[9] Taniguchi Y, Sakakura K, Mukai Y, Yamamoto K, Momomura S-I, Fujita H (2019). Intentional switch between 1.5-mm and 1.25-mm burrs along with switch between rotawire floppy and extra-support for an uncrossable calcified coronary lesion. J Cardiol Cases.

[10] Kirtane AJ, Stone GW (2007). The Anchor–Tornus technique: a novel approach to “uncrossable” chronic total occlusions. Catheter Cardiovasc Interv..

[11] Sakakura K, Taniguchi Y, Yamamoto K, Wada H, Momomura SI, Fujita H (2017). When a burr can not penetrate the calcified lesion, increasing burr size as well as decreasing burr size can be a solution in rotational atherectomy. Int Heart J..

[12] Sakakura K, Ako J, Momomura S (2011). Successful removal of an entrapped rotablation burr by extracting drive shaft sheath followed by balloon dilatation. Catheter Cardiovasc Interv..

[13] Sakakura K, Ako J, Wada H, Naito R, Funayama H, Arao K (2012). Comparison of frequency of complications with on-label versus off-label use of rotational atherectomy. Am J Cardiol.

[14] Sakakura K, Funayama H, Taniguchi Y, Tsurumaki Y, Yamamoto K, Matsumoto M (2017). The incidence of slow flow after rotational atherectomy of calcified coronary arteries: a randomized study of low speed versus high speed. Catheter Cardiovasc Interv..

[15] Sakakura K, Ako J, Wada H, Naito R, Arao K, Funayama H (2012). Beta-blocker use is not associated with slow flow during rotational atherectomy. J Invasive Cardiol..

[16] Matsuo H, Watanabe S, Watanabe T, Warita S, Kojima T, Hirose T (2007). Prevention of no-reflow/slow-flow phenomenon during rotational atherectomy—a prospective randomized study comparing intracoronary continuous infusion of verapamil and nicorandil. Am Heart J.

[17] Barbato E, Carrie D, Dardas P, Fajadet J, Gaul G, Haude M (2015). European expert consensus on rotational atherectomy. EuroIntervention..

[18] Yamamoto K, Sakakura K, Taniguchi Y, Tsurumaki Y, Wada H, Momomura S-I (2018). Trapping balloon technique for removal of the burr in rotational atherectomy. Int Heart J..

[19] Levey AS, Coresh J, Greene T, Stevens LA, Zhang YL, Hendriksen S (2006). Using standardized serum creatinine values in the modification of diet in renal disease study equation for estimating glomerular filtration rate. Ann Intern Med.

[20] Safian RD, Feldman T, Muller DW, Mason D, Schreiber T, Haik B (2001). Coronary angioplasty and rotablator atherectomy trial (CARAT): immediate and late results of a prospective multicenter randomized trial. Catheter Cardiovasc Interv..

[21] Whitlow PL, Bass TA, Kipperman RM, Sharaf BL, Ho KK, Cutlip DE (2001). Results of the study to determine rotablator and transluminal angioplasty strategy (STRATAS). Am J Cardiol.

[22] Tomey MI, Kini AS, Sharma SK (2014). Current status of rotational atherectomy. JACC Cardiovasc Interv..

[23] Kini A, Marmur JD, Duvvuri S, Dangas G, Choudhary S, Sharma SK (1999). Rotational atherectomy: improved procedural outcome with evolution of technique and equipment. Single-center results of first 1000 patients. Catheter Cardiovasc Interv..

[24] Brown CG, Kelen GD, Ashton JJ, Werman HA (1987). The beta error and sample size determination in clinical trials in emergency medicine. Ann Emerg Med.

